# Behavioral disorders as unusual presentation of pediatric extraventricular neurocytoma: report on two cases and review of the literature

**DOI:** 10.1186/s12883-014-0242-8

**Published:** 2014-12-19

**Authors:** Raffaella Messina, Maria Giuseppina Cefalo, Domitilla Elena Secco, Simona Cappelletti, Erika Rebessi, Andrea Carai, Giovanna Stefania Colafati, Francesca Diomedi Camassei, Antonella Cacchione, Carlo Efisio Marras, Angela Mastronuzzi

**Affiliations:** Department of Neuroscience and Neurorehabilitation, Neurosurgery Unit, Bambino Gesù Children’s Hospital, IRCCS, Piazza Sant’ Onofrio 4, 00165 Rome, Italy; Department of Hematology/Oncology and Stem Cell Transplantation, Bambino Gesù Children’s Hospital, IRCCS, Piazza Sant’Onofrio 4, 00165 Rome, Italy; Department of Neurosciences, Neurology Unit, Bambino Gesù Children’s Hospital, IRCCS, Piazza Sant’Onofrio 4, 00165 Rome, Italy; Department of Radiology/Neuroradiology Unit, Bambino Gesù Children’s Hospital, IRCCS, Piazza Sant’ Onofrio 4, 00165 Rome, Italy; Department of Anatomical Pathology, Bambino Gesù Children’s Hospital, IRCCS, Piazza Sant’ Onofrio 4, 00165 Rome, Italy

**Keywords:** Extraventricular neurocytoma, Pediatric brain tumors, Behavioral disorder, Attention deficit/hyperactivity disorders

## Abstract

**Background:**

Extraventricular neurocytomas (EVNs) are rare parenchymal brain tumors, distinct from central neurocytomas that are typically located within the supratentorial ventricular system. Seizures and headache represent the most common symptoms of extraventricular neurocytomas in the cerebral hemisphere both in adult and pediatric population.

**Case presentation:**

We describe two cases of pediatric EVN with clinical onset characterized by behavioral and attention deficit/ hyperactivity disorders. The association between behavioral/attention disorders in childhood and the presence of a frontal neurocytoma has never been described before. Furthermore, inappropriate levels of inattention, hyperactivity and impulsivity are common among the neurobehavioral and developmental disorders in childhood. We reviewed 43 pediatric cases of extraventricular neurocytoma included in the PubMed database and their clinical presentation, and we never found this unusual relationship.

**Conclusion:**

In childhood, the attention/hyperactivity disorders seem to be often over-diagnosed. When these deficits are more subtle and do not well-fit in a specific neurocognitive disorder, the clinicians should have a suspicion that they might mask the clinical features of a frontal lesion. This paper is focused on the clinical presentation of the extraventricular neurocytoma and the possible organic etiology of an attention and hyperactivity deficit.

## Background

Extraventricular neurocytoma (EVN) is a parenchymal brain tumor distinct from central neurocytoma. It is most commonly located in the frontal and parietal lobes [1] and included in the 2007 World Health Organization (WHO) classification of tumors of the central nervous system [2].

Although the incidence of EVN in childhood is not known, it is certainly a rare tumor. It is composed by either glial or neuronal cell differentiation. EVNs have a potential aggressive behavior based on the MIB-1 labeling index (>3%) and on atypical histological features. They are mostly confused with oligodendrogliomas or ependymomas [3]. Clinical symptoms are usually dependent on side and size tumor. Partial seizures and headache represent the most frequent clinical presentation of EVN. Even though the EVNs are commonly located in the frontal lobe, behavioral or cognitive development disorders have never been described as presentation symptoms.

## Methods

Written informed consent was obtained from the patients’ parents for publication of this Case report and any accompanying images. We report on two cases of extraventricular neurocytoma in two children referred to the Neuropsychiatry Unit of Bambino Gesù Children’s Hospital in Rome with the diagnosis of attention deficit/ hyperactivity disorder (ADHD). We further reviewed 43 pediatric cases of EVN included in the PubMed database with particular reference to onset symptoms.

## Case presentation

### Patient 1

**Figure 1:**
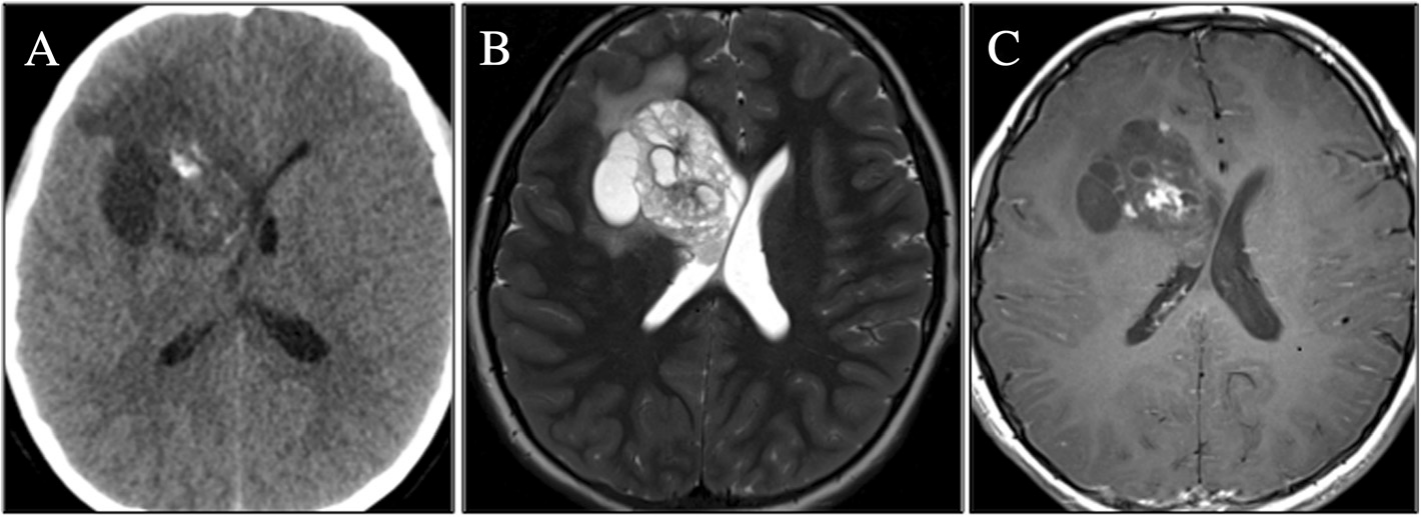
**Patient 1** **:** **head CT scan and brain MRI**, **axial images. (A)** CT scan: large, calcified and heterogeneous round mass in the right frontal lobe, with hyperdense spots and edema surrounding the lesion; **(B)** MR T2 weighted images: enhancing lobulated mass with microcystic and solid components; **(C)** Contrast-enhanced image: inhomogeneous “patchy” enhancement of the lesion.

**Figure 2:**
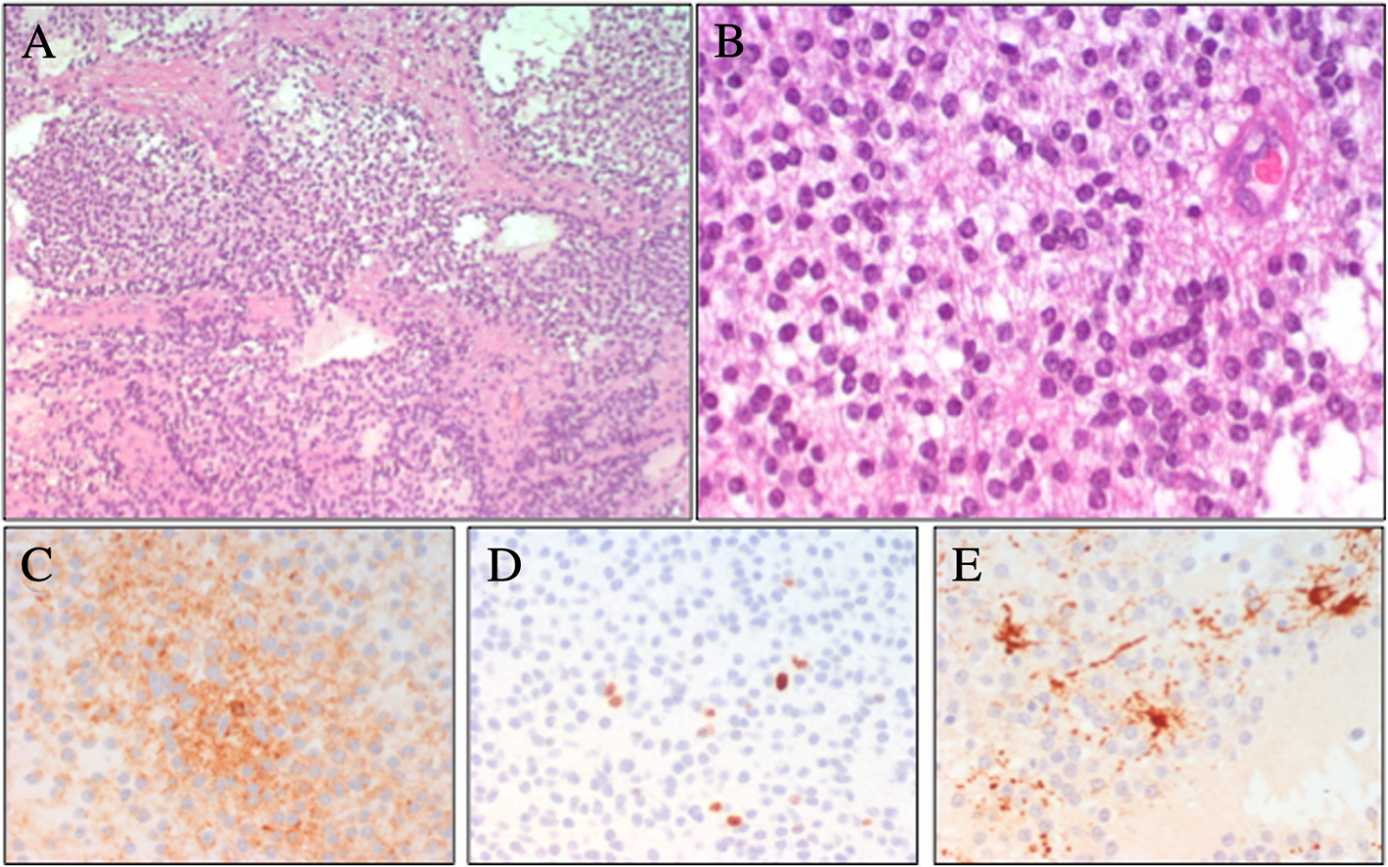
**Patient 1** **:** **photomicrographs of the extraventricular neurocytoma.** Specimens showing: **(A-B)** the uniform population of round cells (hematoxylin and eosin 20× – 40×), with synaptophysin-positive cells **(C)**. Photomicrographs of **(D)** low immunoreactivity to Ki-67 and **(E)** GFAP stain marked scattered reactive astrocytes.

A 10-year-old male, with a clinical diagnosis of ADHD since he was 7-year-old, was admitted to our Hospital with persistent headache and vomiting. Physical examination showed bilateral papilloedema. No other neurological signs were evident. Non-enhanced brain CT scan documented a large (4.5 cm), calcified and heterogeneous round mass in the right frontal lobe with vasogenic edema. Brain MRI confirmed an enhancing lobulated mass with micro-cystic and solid components, causing a right-to-left shift and disappearance of the frontal horn of the right ventricle (Figure 1). The patient underwent a gross total resection of the lesion with a histological diagnosis of ventricular neurocytoma (WHO Grade II). A monomorphous neoplasia composed of small, round synaptophysin-positive cells with uniform distribution was observed, with a very low proliferation rate (1-2%); GFAP staining showed the presence of scattered reactive astrocytes (Figure 2). The patient recovered well, showing a resolution of raised intracranial pressure symptoms and remaining neurologically intact with a progressive reduction of affective problems, attention deficit and anxiety disorders.

### Patient 2

**Figure 3:**
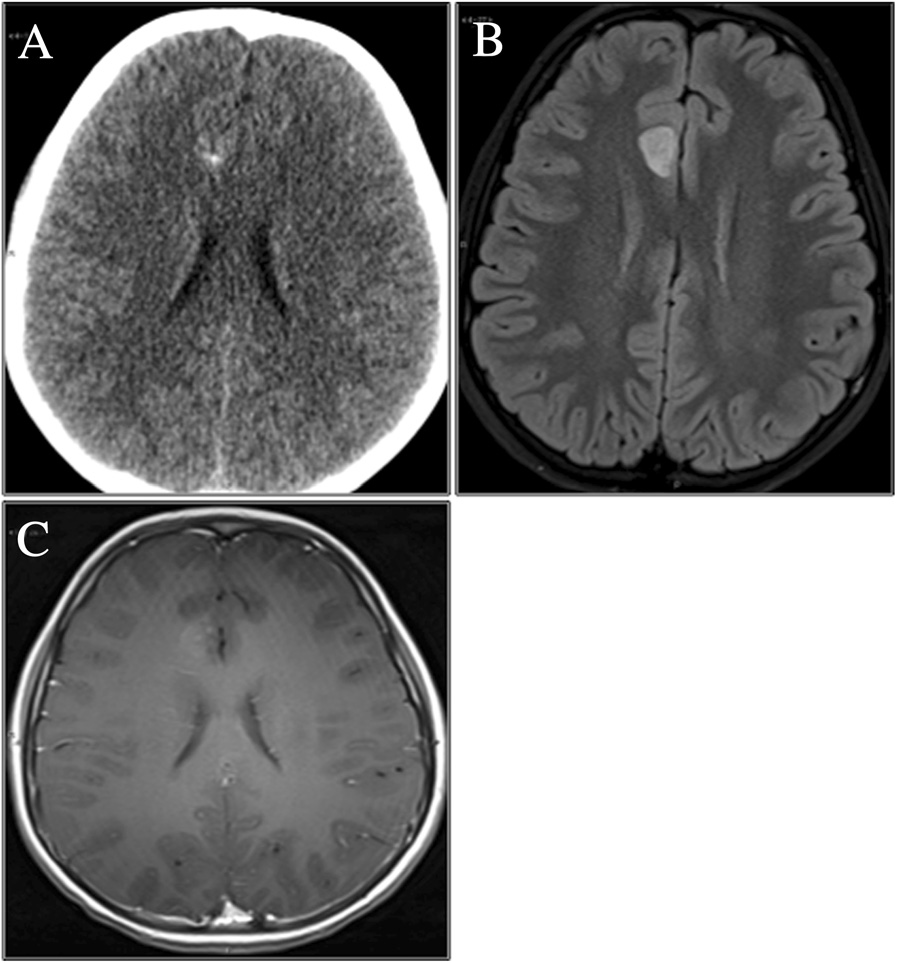
**Patient 2:** **head CT scan and brain MRI**, **axial images. (A)** CT scan: calcified spots inside a right frontomesial mass; **(B)** FLAIR images: parasagittal mass of the right frontal lobe, involving the anterior part of the cingulated gyrus. No perilesional edema; **(C)** T1-weighted image: heterogeneous post-contrast enhancement.

**Figure 4:**
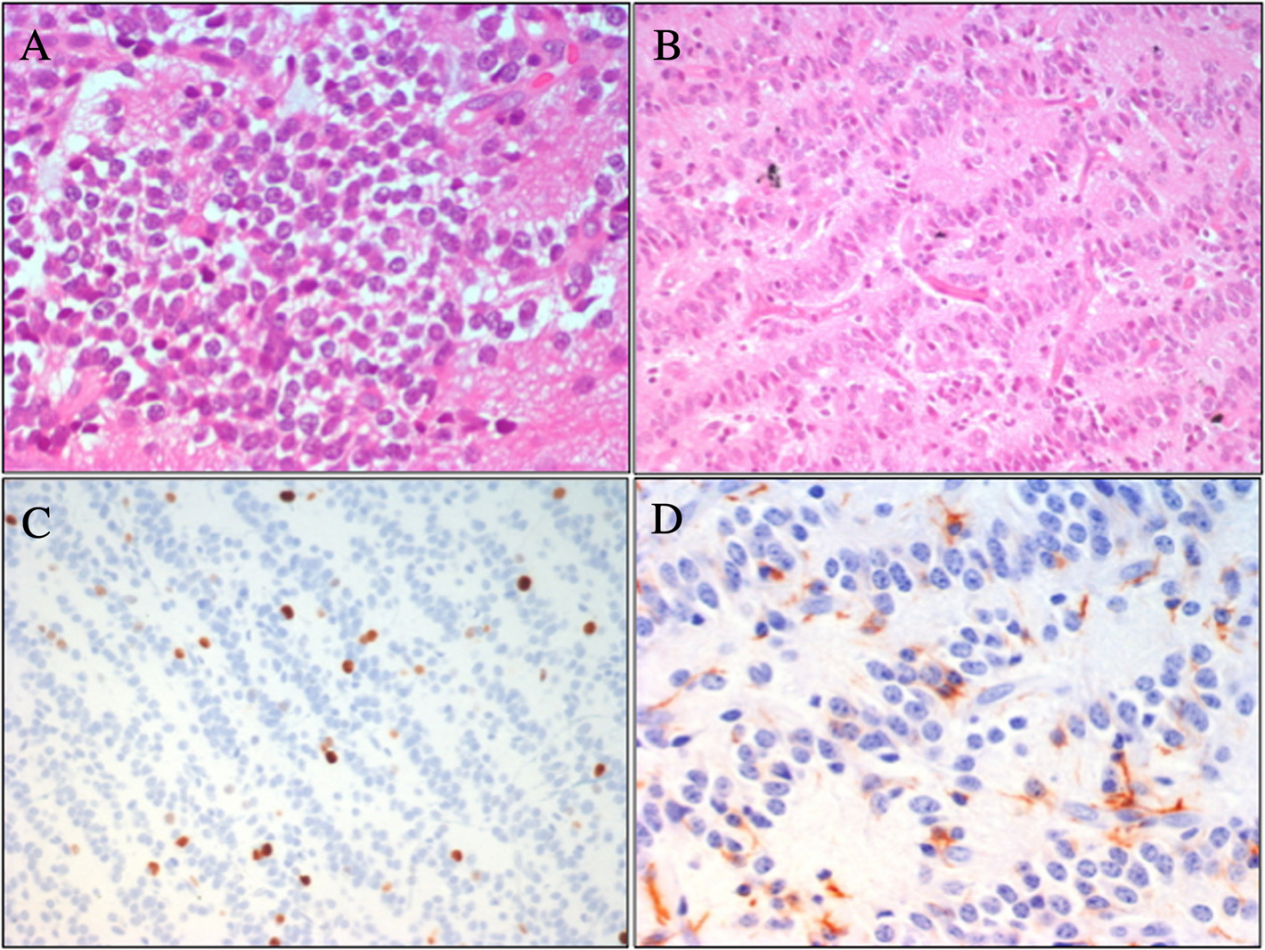
**Patient 2:** **photomicrographs of the extraventricular neurocytoma. (A-B)** Small, round, synaptophysin-positive cells, with a prevalent “ribbon-like” growing pattern and scattered more classical monomorphous areas (hematoxylin and eosin 20x). Photomicrographs of **(C)** high immunoreactivity to Ki-67 and **(D)** GFAP stain marked scattered reactive astrocytes.

A 10-Year-old male was admitted to our Hospital, suffering from frontal headache and focal seizures with a sudden onset. At 7 years a diagnosis of ADHD was performed, based on clinical evidence of speech and learning delay with attention deficit and anxiety symptoms. Head CT scan revealed a right mesial frontal mass, with calcifications. Brain MRI confirmed the small (12 x 17 mm) parasagittal mass of the right frontal lobe, involving the anterior part of the cingulate gyrus. The lesion demonstrated a heterogeneous post-contrast enhancement on T1-weighted image, while restricted diffusion was observed in the solid component (Figure 3). A video-EEG monitoring documented spikes and slow waves, originating from the right frontal area. Treatment with carbamazepine was started. A diagnosis of EVN (grade II WHO) was performed after a gross total resection. The tumor showed proliferation of small, round, synaptophysin-positive cells with a prevalent ribbon-like pattern of growth and scattered more classical monomorphous areas; MIB-1 was about 3-4%. GFAP staining marked scattered reactive astrocytes (Figure 4). After surgery the patient was seizure free with normal EEG and without additional neurological symptoms. The neurocognitive follow up showed a progressive behavioral improvement with a persistence of affective problems but with a reduction of anxiety disorders.

## Discussion

Neurocytoma is a rare tumor of the central nervous system, mainly recognized within the ventricular system adjacent to the Monro’s foramina in middle-aged patients. It displays both neuronal and glial differentiation. Extraventricular neurocytoma was introduced as a separate entity from central neurocytoma in the 2007 World Health Organization (WHO) classification of CNS tumors [2], because of a parenchymal site and a more aggressive neurobiological behaviour. EVN has a marked tendency to glial differentiation [4]. Raised intracranial pressure symptoms are commonly associated to the presence of these lesions, not only due to the size of the lesion but also to the perilesional edema. It can also rarely occur in childhood. Data from the literature about EVNs in the pediatric population, derive almost exclusively from case reports. A literature review about pediatric cases of EVNs included in the PubMed database in the last 20 years identified 29 studies, overall 43 patients [5-8,4,9-14,1,15-31]. Tumor location and presenting symptoms are summarized in Table 1. Lesions were supratentorial in 32 out of 43 cases (74%) and infratentorial in 5 cases (11%). In the remaining six patients (13%) the lesion was located in the spinal cord. Among patients with a supratentorial tumor, EVN was located in the frontal lobe in 20 cases (62.5%), whereas in the remaining 12 patients (37.5%) the tumor location was temporal, parietal, occipital, thalamic or hypothalamic. Age at clinical onset ranged from 1 to 18 years (mean 8.5 yrs). Despite the prevalence of the EVN in the frontal lobe, the seizure activity is the most common presenting symptom, followed by headache and cranial nerve palsy. None of the reported patients had behavioral disorders or ADHD as presenting symptoms. In our patients a diagnosis of ADHD had been made several years earlier than the occurrence of classical symptoms of EVN such headache, vomiting, papilloedema and seizures.

**Table 1:** **Summary of demographic data**, **tumor location and symptoms in pediatric cases of EVNs reported in literature** (**n.d**.: **not defined**)

	**Authors**	**Age** ** (years)**	**Tumor location**	**Presenting symptoms**
**1**	Tortori-Donati P et al. [5]	9	Temporal	Seizures
**2**	Myung JK et al. [6]	9	Frontal	Headache
**3**	Brat DJ et al. [7]	From 5 to 18 (7 patients)	Cerebrum	n.d.
**4**	Limaiem F et al. [8]	4	Cerebral hemisphere	Seizures
**5**	Giangaspero F et al. [4]	From 5 to 18 (4 patients)	Frontal, occipital, temporal, parietal, hypothalamus	Seizures, dizziness
**6**	Agarwal S et al. [9]	16	Spinal cord	n.d.
**7**	Ahmad F et al. [10]	15	Brainstem, cerebellum	Hydrocephalus
**8**	Brandis A et al. [11]	1	Cerebellum	n.d.
**9**	Buchbinder D et al. [12]	1	Frontal	n.d.
**10**	Cheung YK [13]	n.d.	Thalamus	n.d.
**11**	Choi H et al. [14]	8	Frontal	n.d.
**12**	Garber ST and Brockmeyer DL [1]	8	Frontal	Seizures
**13**	Ghosal N et al. [15]	9	Frontoparietal	n.d.
**14**	Hamilton R [16]	11	Frontal	Headache, nausea, vomiting
**15**	Harada M et al. [17]	7	Frontal	Seizures
**16**	Makhdoomi R et al. [18]	5	Cerebellar vermis	Headache, vomiting,
**17**	Möller-Hartmann W et al. [19]	16	Parietooccipital	Headache
**18**	Mpairamidis E et al. [20]	3	Parietotemporal	Seizures
**19**	Nishio S et al. [21]	1 - 2 (2 patients)	Frontal Temporal	Seizures, loss of consciousness, headache
**20**	Pal L et al. [22]	6	Cerebellar	Vomiting, headache, cerebellar symptoms
**21**	Polli FM et al. [23]	6 - 15 (2 patients)	Spinal cord	Chronic intracranial hypertension, paraesthesiae and limb weakness
**22**	Psarros TG et al. [24]	1 yr,3mo	Spinal cord	Somnolence, bilateral sixth nerve palsy, limb weakness
**23**	Raja AI et al. [25]	7	Occipital	NF1
**24**	Singh A et al. [26]	8	Spinal cord	Paraparesis, urinary incontinence
**25**	Stapleton SR et al. [27]	12	Spinal cord	Spinal pain, numbness, paraesthesiae and limb weakness
**26**	Treier M et al. [28]	11	Temporal	Seizures
**27**	Yang GF et al. [29]	2	Frontal	Hemiparesis
**28**	Yi KS et al. [30]	from 10 to 13 (4 patients)	Cerebral hemisphere, cerebellum and thalamus	Headache, dizziness, seizures
**29**	Han et al. [31]	2	Frontal	Vomiting and papilloedema

Attention deficit/hyperactivity disorder has to be well defined among the behavioral disorders appearing at school age, in order to avoid misdiagnosis. ADHD defines significant and wide-ranging impairments and affects as many as 3 to 9% of children worldwide. It is based on strict criteria but is often over-diagnosed. The diagnosis of primary ADHD is made by DSM IV criteria when there is no evidence from the history, the physical examination or laboratory findings of any other condition producing a similar clinical picture. Other psychiatric and neurological disorders such as traumatic brain injury, epilepsy and depression can lead to disturbances in attention and/or activity level. In patients with frontal tumor masses and low proliferation index, the attention impairment and behavioral disorders probably result from direct disruption of frontal lobe functioning or disconnection from subcortical afferents.

**Figure 5:**
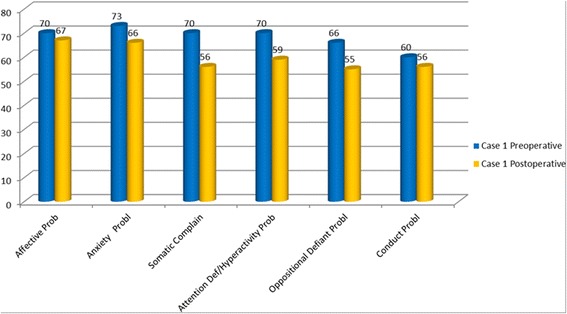
**DSM**-**Oriented scales of the Child Behavior Checklist** **(CBCL):** **preoperative and postoperative assessment in the patient 1.** T Score ≤60 Range Normal; 61-70 Range Borderline; >70 = Range Clinical.

**Figure 6:**
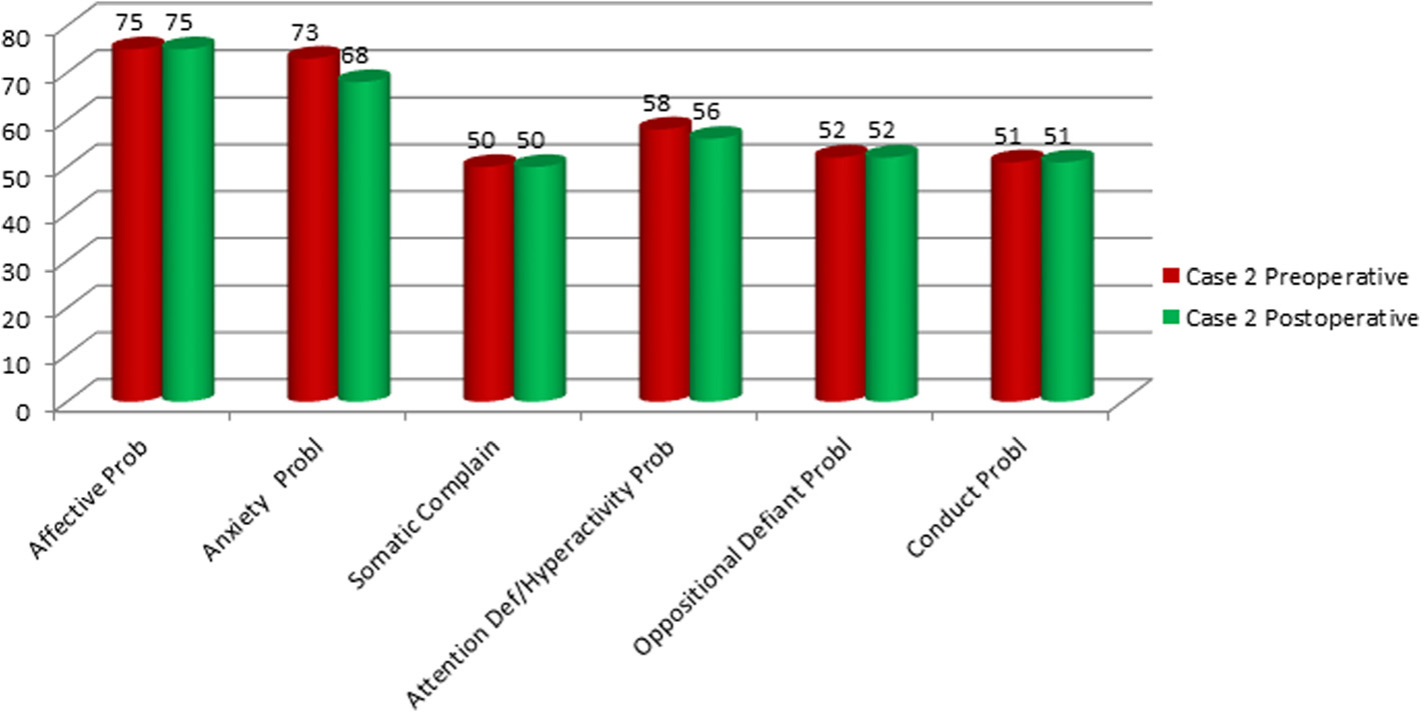
**DSM-** **Oriented scales of the Child Behavior Checklist (** **CBCL):** **preoperative and postoperative assessment in the patient 2.** T Score ≤60 Range Normal; 61-70 Range Borderline; >70 = Range Clinical.

Our patients were evaluated with cognitive and behavioral assessment at the time of admission, before and after surgery, with one-year follow-up intervals. Clinical history reported by parents showed that language and motor delay had already been present since they were 4-year-old. Therefore we hypothesized a delayed global development, overlooked and underestimated when the ADHD was diagnosed. Cognitive assessment was performed by Wechsler Intelligence Scales for Children (WISC III), documenting a normal range and no significant difference between verbal and performance skills. Behavioral symptoms were clinically investigated and standardized by Child Behavior Checklist (CBCL 6-18) DSM IV oriented, used for children and teenager [32]. Patient 1, preoperatively evaluated, showed an affective (T score = 70) and attention impairment (T score = 70) and a significant anxiety problem (T score = 73). Postoperative assessment, 14 months after surgery, showed no evidence of symptoms previously reported: affective (T score = 67) and attention impairment (T score = 59) and anxiety problem (T score = 66) (Figure 5). Patient 2 showed before surgery affective (T score = 75) and anxiety problem (T score = 73); 12 months after surgery there was a persistence of a affective problems (T score = 75) and a lowering of anxiety problem (T score = 68) (Figure 6). Both patients showed an affective and attention impairment and significant anxiety. Based on these evaluations a diagnosis of ADHD could not be confirmed. Previous formal testing for ADHD diagnosis was not available at the time of admission in the described cases.

Patient 2 had focal seizures shortly before diagnosis and they stopped after surgery, with normal EEG pre and post-operatively. Moreover, the progressive improvement after tumor resection suggests a frontal lobe disfunction as the origin of observed neuro-behavioural symptoms.

Attention deficit hyperactivity disorder is associated with multiple neuropsychological deficits. Several studies underline how difficult it is to make a diagnosis of ADHD. Our experience suggests caution in cases with significant comorbidity before excluding alternative diagnoses.

A recent study, based on electronic records of Emergency Department visits for 87 pediatric patients with brain tumors, retrospectively reviewed from 2002 to 2011, shows that behavior/school change and altered mental status appear with a low frequency, less than 17% [33]. These data are probably justified by brain plasticity during childhood allowing good compensation of neuro-cognitive impairment and resulting in intracranial hypertension as the most frequent symptom at diagnosis.

Our patients underwent gross total resection. Surgical resection remains the main management strategy for a good outcome. When a subtotal surgical resection is performed, radiotherapy could be considered to ensure better local disease control.

Neuropsychological follow-up one year after resection showed progressive behavioral improvement in both patients, suggesting that the initial diagnosis was not ADHD.

## Conclusions

Attention deficit in childhood, not symptomatic of other injuries, do not tend to improve with age, while an age-related improvement is possible in hyperactivity disorders. We ascribe the improvement of the behavioural performance to the surgical resection, in our patients. This suggests that the attention deficit/hyperactivity disorder was linked to the presence of the EVN. Our report should stimulate clinicians to be careful to the behavioral disorders in childhood not connectable to a clear neuropsychological diagnosis, such as a poor academic achievement and problematic peer relationship, since they could have an organic etiology.

### Consent

Written informed consent was obtained from the patients’ parents for publication of these Case reports and any accompanying images. A copy of the written consent is available for review by the Editor of this journal.

## Abbreviations

EVN: Extra ventricular neurocytoma
ADHD: Attention deficit/hyperactivity disorder
WISC III: Wechsler intelligence scales for children
CBCL: Child behavior checklist
WHO: World Health Organization
